# Simultaneous Determination of Seven Components in Rat Plasma by the UPLC-MS/MS Method and Application of Pharmacokinetic Studies to SimiaoYong’an Decoction

**DOI:** 10.3390/molecules22111937

**Published:** 2017-11-09

**Authors:** Yuanyan Liu, Sensen Chi, Weihua Wang, Lei Su, Bin Liu

**Affiliations:** 1 School of Chinese Pharmacy, Beijing University of Chinese Medicine, No. 6, Beijing Central South Road, Chaoyang District, Beijing 100102, China; yyliu_1980@163.com (Y.L.); 17801085298@163.com (S.C.); lei_susu@126.com (L.S.); 2 Chemical Metrology & Analytical Science Division, National Institute of Metrology, P.R., No. 18, EastRoad of the Third North Circle Ring, Chaoyang District, Beijing 100013, China; wangwh@nim.ac.cn

**Keywords:** SimiaoYong’an Decoction, UPLC-MS/MS, simultaneous determination, pharmacokinetics

## Abstract

SimiaoYong’an Decoction (SYD) is a classical traditional Chinese prescription that is used for the treatment of gangrene, heat-clearing, detoxification and pain alleviation. We developed a sensitive ultra-performance liquid chromatography-tandem mass spectrum (UPLC-MS/MS) method for the simultaneous determination of seven major active ingredients of SYD extract (i.e., harpagide, chlorogenic acid, sweroside, loganin, liquiritin, angoroside C and harpagoside) in rat plasma. The preliminary steps in the plasma analysis were the addition of an internal standard such as linarin, followed by protein precipitation with methanol. Separation of the active ingredients was performed on an ACQUITY UPLC^®^ BEH C18 column (100 mm × 2.1 mm, 1.7 μm) at a flow rate of 0.2 mL/min with methanol/water 0.1% formic acid aqueous (*v*/*v*) as the mobile phase. Detection was performed on a triple quadrupole tandem MS (QqQ-MS) via negative ion electrospray ionization in multiple reactions monitoring (MRM) mode. All calibration curves showed good linearity (r > 0.99) over the concentration range with a low limit of quantification between 0.029 and 5.813 ng/mL. Precision was evaluated by intra-day and inter-day assays, and the percentages of the RSD were all within 8.1%. The extraction efficiency and matrix effect were 80.6–113.6% and 82.9–99.5%, respectively. The validated method was successfully applied to a pharmacokinetic study in rats after oral administration of SYD extract and the corresponding single and combined traditional Chinese medicines (TCMs). The pharmacokinetic properties of the seven ingredients showed dynamic changes due to counteraction among the different coexisting components. The established approach has proven useful in the study of the active constituents in a traditional Chinese prescription.

## 1. Introduction

SimiaoYong’an Decoction (SYD) is a classical Chinese prescription that is used for the treatment of gangrene, heat-clearing, detoxification and pain alleviation. SYD was firstly recorded in “Hua Tuo Shen Yi MiZhuan” in the Han dynasty and then listed in BaoXiangdun’s “Yan Fang Xin Bian” in the Qing dynasty [1,2,3]. It is composed of four single herbs, which are *Lonicera japonica* Thunb. (LJ), *Scrophularia ningpoensis* Hemsl. (SN), *Angelica sinensis* (Oliv.) Diels (AS) and *Glycyrrhiza uralensis* Fisch. (GU). LJ is commonly used as a heat-clearing and detoxifying agent and is applied as a monarch drug in this prescription. SN, which is used as an adjuvant drug, shows the property of clearing away heat in the kidneys and purging fire for removing toxin functions. AS is used as a complement drug with the property of nourishing Yin, promoting blood circulation, and moisturizing dryness functions. The mediator drug GU exhibits the functions of invigorating the spleen and replenishing Qi, expelling phlegm and relieving cough, relieving spasm and pain, as well as coordinating with other herbs. The above four herbs combined make up a classic prescription that is used to treat gangrene disease. Additionally, in modern pharmacological studies, SYD was proven to cure heat-toxin thromboangiitis obliterans and vascular thrombosis diseases of different origin [4,5].

Until now, many types of compounds have been reported in herbs composing SYD [6,7,8,9,10,11], including flavonoids (i.e., liquiritin), iridoids (i.e., harpagide, sweroside, loganin and harpagoside), phenylpropanoids (i.e., angoroside C and chlorogenic acid), terpenoids and others. Among them, chlorogenic acid and liquiritin exhibit various pharmacological activities including antioxidative, antiviral, antibacterial, anti-inflammatory, anti-endotoxin, antitumor and liver protective effects [12,13,14]. Representative iridoids such as harpagide, sweroside, loganin and harpagoside have been reported to exhibit anti-inflammatory, antipyresis, analgesia, anti-virus and liver protection effects [15,16]. Angoroside C has been shown to have an anti-fatigue effect [17].

Numerous pharmacokinetic studies have focused on LJ, SN, AS and GU as single herbs. For instance, Zhou et al. [18] established an HPLC-MS/MS method for pharmacokinetic and absolute bioavailability studies of chlorogenic acid from LJ in rats with different administration routes. Luo et al. [19] developed a specific UPLC-MS/MS for the pharmacokinetic study and tissue distribution of AS coupled with other herbs. Li et al. established and applied a new LC-MS/MS method for analyzing the metabolism, distribution, excretion and attenuation mechanism of triptolide from GU in rats [20]. HPLC-MS and pharmacokinetic studies referred to harpagide [21], chlorogenic acid [18], sweroside [22], loganin [23], liquiritin [24], angoroside C [25] and harpagoside [25] have been reported.

Although pharmacokinetic studies based on the single herb of SYD have been reported, data on the pharmacokinetics of the total extract of SYD are limited. In the present study, seven characteristic compounds from three chemical types, such as flavonoids (i.e., liquiritin), iridoids (i.e., harpagide, sweroside, loganin and harpagoside) and phenylpropanoids (i.e., angoroside C and chlorogenic acid), were selected as reference analytes for integral pharmacokinetic studies of SYD. Here, a sensitive and selective UPLC-MS/MS method for the simultaneous determination of seven reference analytes is reported. The established methodology was applied to investigate the pharmacokinetic characteristics of these compounds following oral administration of extracts of SYD and its single and combined TCMs to rats.

## 2. Results and Discussion

### 2.1. Parameter Optimization of UPLC-MS/MS

The UPLC-MS/MS method described here was developed to facilitate the simultaneous quantification of harpagide, chlorogenic acid, sweroside, loganin, liquiritin, angoroside C, harpagoside and IS in rat plasma. Due to the diverisities of analytes and their wide range of solubilities, chomatographic parameters such as column type, mobile phase composition, gradient elution procedure, flow rate of the mobile phase and column temperature were optimized to obtain suitable chromatographic separation and ionization (shown in Section 3.3). To fulfill the simultaneous determination, separation of the seven analytes was performed on an ACQUITY UPLC^®^ BEH C18 column (100 mm × 2.1 mm, 1.7 μm) at a flow rate of 0.2 mL/min with methanol/water 0.1% formic acid aqueous (*v*/*v*) as the mobile phase.

The water mobile phase of the UPLC-MS/MS system was spiked with 0.1% formic acid to improve ionization. The ionization of these seven reference compounds wasinvestigated in both positive and negative ion mode.The observed response of the analytes was better in the negative ESI mode than in the positive ESI mode. All analytes, the IS included, were quantified in MRM mode at *m*/*z* transitions of 183.11 for harpagide, 191.05 for chlorogenic acid, 125.07 for sweroside, 227.12 for loganin, 255.13 for liquiritin, 175.05 for angoroside C, 147.03 for harpagoside and 283.15 for the IS. The fragmentor was also optimized to enhance the signal response of each standard and to eliminate interference from noise. All factors related to MS performance includingionization mode, capillary voltage, fragmentor voltage, collisionenergy, and gas flow have been optimized as described in Table 1. These conditions were found to be optimal for the determination of all analytes.

### 2.2. Method Validation

#### 2.2.1. Selectivity

**Figure 1:**
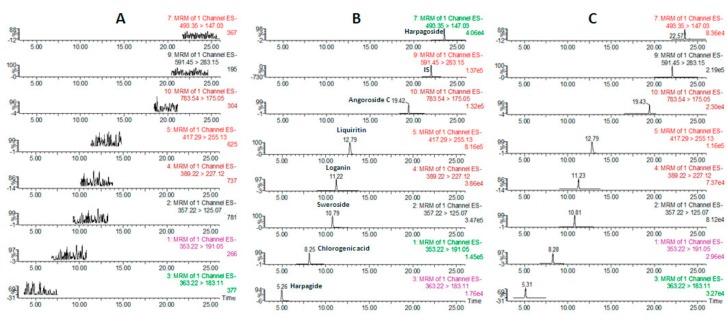
Chromatograms of the analytes and IS in blank plasma (**A**), blank plasma spiked with the analytes and IS (**B**) and a plasma sample from a rat orally administered SimiaoYong’an Decoction(SYD) extract (**C**).

Figure 1 shows the UPLC-MS/MS chromatograms of blank plasma (A), blank plasma spiked with a solution containing all of the analytes as well as the IS in lower limit of quantification(LLOQ) (B) and a plasma sample at 60 min after oral administration of SYD extract (C). The retention times for harpagide, chlorogenic acid, sweroside, loganin, liquiritin, angoroside C, harpagoside and IS were 5.73, 8.79, 11.12, 11.37, 13.54, 19.92, 23.76, and 22.58 min, respectively, as shown in Table 1. High resolution (Rs > 1.5) among the peaks was achieved, and no interfering peak in the blank plasma was observed under these experimental conditions.

#### 2.2.2. Linearity and LLOQ

Typical equations for the calibration curves and correlation coefficients (r) were as follows: *y* = 0.0045*x* − 0.0012 (r = 0.999) for harpagide, *y* = 0.0764*x* − 1.0344 (r = 0.998) for chlorogenic acid, *y* = 0.0026*x* + 0.7098 (r = 0.998) for sweroside, *y* = 0.0009*x* + 0.0611 (r = 0.997) for loganin, *y* = 0.5082*x* − 0.8751 (r = 0.999) for liquiritin, *y* = 0.0669*x* − 0.1995 (r = 0.998) for angoroside C and *y* = 0.0509*x* + 0.1449 (r = 0.993) for harpagoside. The calibration curves of the seven analytes were linear in the ranges of 0.667–1558.660 ng/mL for harpagide, 0.807–1886.321 ng/mL for chlorogenic acid, 5.813–13,580.340 ng/mL for sweroside, 1.690–3948.969 ng/mL for loganin, 0.161–1003.754 ng/mL for liquiritin, 0.121–2157.445 ng/mL for angoroside C and 0.029–580.335 ng/mL for harpagoside. The LLOQs were the lowest concentration in the range of each analyte, which was sufficient for pharmacokinetic study of the seven analytes following oral administration of SYD extract.

#### 2.2.3. Precision and Accuracy

The results of the precision and accuracy at three concentration levels are summarized in Table 2. The intra-day and inter-day precision for all analytes was less than 8.1%, and the accuracy ranged from 91.4% to 110.0%.

#### 2.2.4. Matrix Effect and Extraction Recovery

Data on extraction recovery and matrix effects are shown in Table 2. The matrix effects of harpagide, chlorogenic acid, sweroside, loganin, liquiritin, angoroside C, harpagoside and SI at three different concentrations from rat plasma were found to be 80.6–111.9%, 81.1–113.6%, 82.9–105.9%, respectively. The extraction recovery of harpagide, chlorogenic acid, sweroside, loganin, liquiritin, angoroside C, harpagoside and SI at three different concentrations from rat plasma was found to be 84.9–97.4%, 82.9–99.5%, 86.8–98.2%, respectively, which indicates good reproducibility that meets the requirements of biological sample analysis.

#### 2.2.5. Stability

The stability of all analytes was evaluated under various conditions. The results listed in Table 2 indicate that the seven analytes were stable in the plasma samples for 4 h at room temperature, for 14 days at −20 °C, through three freeze-thaw cycles and in the auto-sampler at 4 °C for 24 h.

### 2.3. Pharmacokinetic Analysis

**Figure 2:**
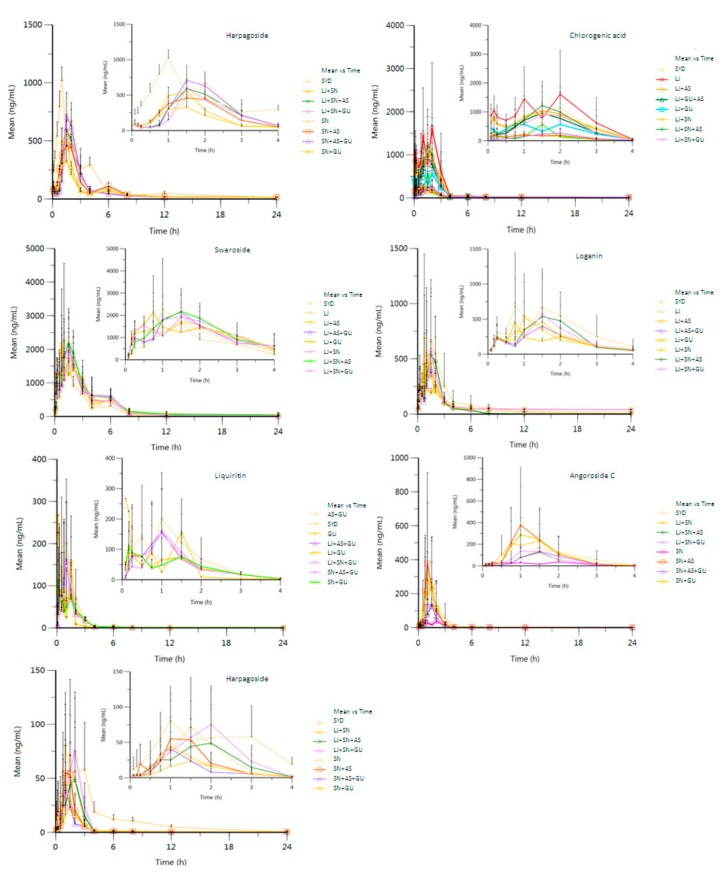
Plasma concentration-time curve of the seven compounds in the fifteen groups of compatibility combinations (mean ± SD, *n* = 6 replicates).

The validated UPLC-MS/MS method was successfully applied to pharmacokinetic studies of harpagide, chlorogenic acid, sweroside, loganin, liquiritin, angoroside C and harpagoside in plasma samples obtained from SD rats that had been orally administered 15 groups of extracts of SYD and its single and combined TCMs (LJ, SN, AS, GU, LJ + SN, LJ + AS, LJ + GU, SN + AS, SN + GU, AS + GU, LJ + SN + AS, LJ + SN + GU, LJ + AS + GU, SN + AS + GU and SYD). The mean plasma concentration-time profiles of harpagide, chlorogenic acid, sweroside, loganin, liquiritin, angoroside C and harpagoside are shown in Figure 2. The corresponding estimated pharmacokinetic parameters are given in Table 3. The assay was sensitive for the determination of the seven reference compounds in plasma samples. Statistical analysis of the pharmacokinetic parameters (*t*_1/2_, *T*_max_, *C*_max_, AUC(0–t), AUC(0–∞), MRT(0–*t*), MRT(0–∞)) was performed with SAS (Statistical Analysis System) 8.2 statistical software through the Student-Newman-Keuls test (*q*-test).

The *T*_max_ values for harpagide (2.00 ± 0.00 h) and chlorogenic acid (1.67 ± 0.26 h) were markedly (*p* < 0.01) increased in SYD compared to other drug combination groups, indicating that the interaction of the compounds may alleviate, eliminate or promote absorption of the given references by affecting the activity of metabolizing enzymes and the expression of the drug transporter in rats. The absorption process is mainly through the paracellular divergence of unsaturated transport. Meanwhile, transcellular transport is accompanied by the dynamic processes of absorption-reabsorption and secretion [26]. It is speculated that there may be some interactions among massive coexisting components, which may change the permeability of the cell membrane or polymerization with the given compound to prolong or shorten the time to reach peak plasma concentration. Harpagoside and cinnamic acid are mainly contained in the extract of SN. Meanwhile, harpagoside tends to produce cinnamic acid by breaking down the ester linkage in vivo. So the plasma pharmacokinetics of these two compounds should be evaluated by considering the transformation process.

The pharmacokinetic parameters of sweroside exhibited a decrease to some extent with SYD compared with other drug combination groups, such that the *t*_1/2_ was 4.14 ± 1.46 h, T_max_ was 1.33 ± 0.49 h, *C*_max_ was 1.97 ± 0.70 μg/mL, AUC(0–*t*) was 4.49 ± 0.28 h μg/mL (*p* < 0.05), AUC(0–∞) was 4.53 ± 0.29 h μg/mL (*p* < 0.01), MRT(0–*t*) was 3.30 ± 0.39 h, and MRT(0–∞) was 3.53 ± 0.29 h. It indicated that the elimination of sweroside was significantly accelerated in SYD over that in other combinations due to a synergic effect among the four herbs. 

The *C*_max_ and AUC(0–*t*) of loganin were 0.77 ± 0.46 μg/mL and 1.98 ± 1.38 h μg/mL, respectively. These two parameters were significantly greater than those in other combination groups. This result shows that the absorption or the bioavailability of loganin was improved in SYD, whereas the MRT(0–*t*) (4.71 ± 1.34 h, *p* < 0.01) and MRT(0–∞) (8.87 ± 8.10 h, *p* < 0.01) were drastically lower in SYD than in the other combined groups, showing that loganin was rapidly eliminated in the liver to counteract the obvious nephrotoxicity of loganin [27]. Thus, the synergistic effect in SYD would be beneficial to alleviate potential renal injury.

The MRT(0–*t*) (2.89 ± 0.92 h) of liquiritin was distinctly increased (*p* < 0.01) in SYD over that in other combined groups. The AUC(0–*t*) and AUC(0–∞) of liquiritin were 0.29 ± 0.21 h μg/mL and 0.34 ± 0.21 h μg/mL, respectively, which are higher than those of other combined groups. Liquiritin as a flavonoid glycoside is an antibacterial ingredient in SYD, and it can be hydrolyzed in the intestine [28]. The synergistic effect among massive compounds in SYD finally promotes absorption and reduces elimination, thus improving the bioavailability of liquiritin.

The *T*_max_ of angoroside C (1.38 ± 0.44 h) in SYD was higher than that in other combined groups, suggesting that its absorption was promoted by counteraction among the massive ingredients. The pharmacokinetic parameters of harpagoside in SYD showed scattered distribution with *t*_1/2_ of 11.20 ± 2.64 h, *T*_max_ of 1.50 ± 0.00 h, *C*_max_ of 0.07 ± 0.07 μg/mL, AUC(0–*t*) to be 0.12 ± 0.09 h μg/mL, AUC(0–∞) to be 0.14 ± 0.09 h μg/mL, MRT(0–*t*) of 3.92 ± 1.31 h, and MRT(0–∞) of 19.81 ± 28.74 h.

As described in the present study, the pharmacokinetic parameters of some ingredients were changed when different ingredients were combined then dosed in rats. First of all, SYD is a classical Chinese prescription that is composed of four single herbs. Different combined groups of the four herbs match along with different co-existent ingredients. Various co-existent ingredients may infect the solubility of the selected analyte, and then change the pharmacokinetic parameters. Second, the global absorption and in vivo metabolism after oral administration were mainly mediated by intestinal microbiota in the gut. The gastrointestinal microbiota can be considered as a site with huge biotransformational capacity of drug, which could be affected by different co-existent ingredients [29]. Finally, CYP in the liver and other tissues are a major source of variability in drug pharmacokinetics and response.For instance, the CYP enzymes have indispensable functions in drug metabolism; also, some ingredients may have effects on CYP enzymatic activity [30,31]. It indicated that the pharmacokinetic study of single TCMs could not represent the integral Chinese prescription. The synergistic effect among massive ingredients in SYD finally affected the pharmacokinetic parameters of some active ingredient and then may promote bioavailability of the holistic prescription.

## 3. Materials and Methods

### 3.1. Chemicals and Standard Substances

#### 3.1.1. Chemicals and Reagents

**Figure 3:**
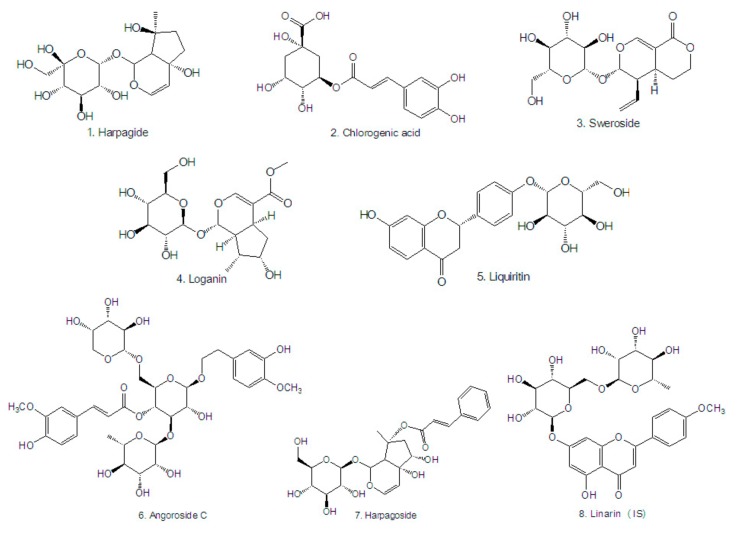
Chemical structures of the analytes and IS.

Acetonitrile and methanol of HPLC grade were obtained from Merck (Darmstadt, Germany). Formic acid was purchased from Sigma-Aldrich (St. Louis, MO, USA). All aqueous solutions were prepared with ultra-pure water produced from a Milli-Q High Purity Water System (Millipore, Billerica, MA, USA). Other reagents were of analytical grade or higher. Reference standards (purity >98%, Figure 3) of chlorogenic acid (batch No.: 110753-201314), sweroside (batch No.: 111742-201101), loganin, (batch No.: 111640-201005), liquiritin (batch No.: 111610-201106), harpagoside (batch No.: 110786-200503) and linarin (internal standard, IS, batch No.: 110715-200512) were purchased from the National Institutes for Food and Drug Control (Beijing, China). Reference standards (purity > 98%, Figure 3) of harpagide (batch No.: 151221) and angoroside C (batch No.: 16100905) were purchased from Chengdu Pufei De Biological Technology Co., Ltd. (Chengdu, China).

#### 3.1.2. Preparation of Standard Solutions and Quality Control (QC) Samples

Primary stock solutions of the seven standard components (harpagide, chlorogenic acid, sweroside, loganin, liquiritin, angoroside C and harpagoside) and the IS were prepared in methanol-water (1:1, *v*/*v*). A mixed stock solution was then obtained by mixing 7.589 μg/mL of harpagide, 9.269 μg/mL of chlorogenic acid, 65.461 μg/mL of sweroside, 18.717 μg/mL of loganin, 1.741 μg/mL of liquiritin, 1.389 μg/mL of angoroside C and 0.292 μg/mL of harpagoside. The IS solution was prepared to a final concentration of 0.247 μg/mL in methanol–water (1:1, *v*/*v*). A series of working standard solutions was prepared by successive dilution of the mixed stock solution with methanol–water (1:1, *v*/*v*). Calibration standards and QC samples were also prepared. For both the calibration standards and QC samples, 50 μL of standard working solution was spiked into 200 μL of blank rat plasma. The calibration concentration ranges were 0.667 ng/mL to 1558.660 ng/mL for harpagide; 0.807 ng/mL to 1886.321 ng/mL for chlorogenic acid; 5.813 ng/mL to 13,580.340 ng/mL for sweroside; 1.690 ng/mL to 3948.969 ng/mL for loganin; 0.161 ng/mL to 1003.754 ng/mL for liquiritin; 0.121 ng/mL to 2157.445 ng/mL for angoroside C; and 0.029 ng/mL to 580.335 ng/mL for harpagoside. In the same manner, the QC samples were prepared at concentrations of 36.021, 346.908 and 1012.666 ng/mL for harpagide; 61.600, 593.260 and 1731.796 ng/mL for chlorogenic acid; 119.394, 1149.863 and 3356.586 ng/mL for sweroside; 35.670, 343.533 and 1002.812 ng/mL for loganin; 31.832, 306.572 and 894.920 ng/mL for liquiritin; 27.215, 262.099 and 765.098 ng/mL for angoroside C; and 12.628, 121.615 and 355.008 ng/mL for harpagoside. All solutions were stored at 4 °C in the refrigerator and brought to room temperature before use.

### 3.2. Preparation of SYD and Its Single and CombinedExtract

Pieces of LJ, SN, AS and GU were weighed according to the proportion of 3:3:2:1 with appropriate amounts. The mixed pieces were crushed and decocted two times (60 min each time) with water (1:10 *w*/*v*) after being soaked for 60 min. The decoctions were then combined and condensed to the concentration of 0.5 g/mL, after which 95% ethanol was slowly added to the concentrate until a final ethanol concentration of 70% was reached. The SYD solution was set at room temperature overnight and then was filtered. The solvent was recovered, and the residue was dried under reduced pressure. A certain amount of the water extract of SYD was weighed and then dissolved with purified water for intragastric (oral) administration solution. The single herbs and different combination groups such as LJ, SN, AS, GU, LJ + SN, LJ + AS, LJ + GU, SN + AS, SN + GU, AS + GU, LJ + SN + AS, LJ + SN + GU, LJ + AS + GU and SN + AS + GU were prepared according to the procedure of SYD. The dose was listed in Table 4.

### 3.3. Animals and Drug Administration

Male Sprague-Dawley (SD) rats (body weight 250 ± 10 g) were supplied by the SPF (Beijing) Biotechnology Co., Ltd. (Certificate No.: SCXK 2016-0002, Beijing, China). The animals were maintained in controlled conditions (temperature 20–25 °C, relative humidity 55–60% and 12-h light/12-h dark cycle) with free access to standard laboratory food and water for seven days of acclimation and then fasted for 12 h before each experiment but allowed to drink freely during this time.

Ninety male SD rats were randomly separated into 15 groups and administered a dosage of extract via gastric gavage once per day; detailed information about the oral daily dose of SYD extract and the corresponding single and combined TCMs (LJ, SN, AS, GU, LJ + SN, LJ + AS, LJ + GU, SN + AS, SN + GU, AS + GU, LJ + SN + AS, LJ + SN + GU, LJ + AS + GU, SN + AS + GU and SYD) is listed in Table 4.

### 3.4. Plasma Sample Pretreatment

Each plasma sample (200 μL) with 50 μL IS solution added was spiked into 100 μL 1% formic acid distilled water solution and then vortexed for 1 min. Protein was precipitated by adding 600 μL of methanol to the mixture. After being vortexed and sonicated, the samples were centrifuged at 25,000 r/min, 4 °C for 10 min. The supernatant of each sample was transferred to a new 2-mL centrifuge tube and evaporated to dryness by N_2_ at 40 °C. Finally, the residue was dissolved in 100 μL methanol–water (1:1, *v*/*v*), followed by centrifugation at 30,000 r/min, 4 °C for 10 min, and 10-μL supernatant aliquots were injected into the UPLC-MS/MS system for analysis.

### 3.5. UPLC-MS/MS Conditions

The UPLC-MS/MS analyses were carried out using a system that consisted of an ACQUITYTM UPLC system (Waters, Milford, MA, USA) in which the power was coupled to a Waters ACQUITY triple quadrupole mass spectrometer(QqQ-MS)equipped with a Z-spray electrospray ionization (ESI) source. The ionization of the 7 reference compounds and the IS was investigated both in positive and negative ESI ion modes. Then, the negative ion mode was selected. All analytes, the IS included, were quantified in MRM mode at *m*/*z* transitions of 363.22 to 183.11 for harpagide, 353.22 to 191.05 for chlorogenic acid, 357.22 to 125.07 for sweroside, 389.22 to 227.12 for loganin, 417.29 to 255.13 for liquiritin, 783.54 to 175.05 for angoroside C, 493.35 to 147.03 for harpagoside and 591.45 to 283.15 for the IS. The parameters of the mass spectrometer were set as follows: Capillary voltage, 3 kV; source temperature, 500 °C; desolvation gas (N_2_) flow rate, 1000 L/h; desolvation gas (N_2_) temperature, 500 °C; and collision gas (Ar) flow rate, 0.15 mL/min^−1^. The parameters for all analytes were tested and the IS of the mass spectrometer were optimized (Table 1). The operation of the UPLC-MS/MS system and data analysis were performed with the MassLynxTM V4.1 workstation (Micromass, Manchester, UK).

LC analyses were performed in gradient elution mode using an ACQUITY UPLC^®^ BEH C18 column (100 mm × 2.1 mm, 1.7 μm, Waters, Milford, MA, USA) equipped with a guard column at 30 °C. The mobile phase consisted of 0.1% formic acid water solution (A) and methanol (B). A linear gradient at a flow rate of 0.2 mL/min was run at 10–15% B for 0–5 min, 15–33% B for 5–10 min, 33% B for 10–15 min, 33–50% B for 15–20 min, 50–75% B for 20–25 min, and 75–100% B for 25–30 min. The samples were kept at 4 °C in the auto-sampler, and a volume of 10 μL was injected into the UPLC system.

### 3.6. Method Validation

The UPLC-MS/MS method was fully validated in terms of selectivity, linearity, LLOQ, accuracy and precision, extraction recovery and matrix effect and stability according to the U.S. Food and Drug Administration (FDA) guidelines for the validation of bioanalytical methods [32,33].

**Figure 4:**
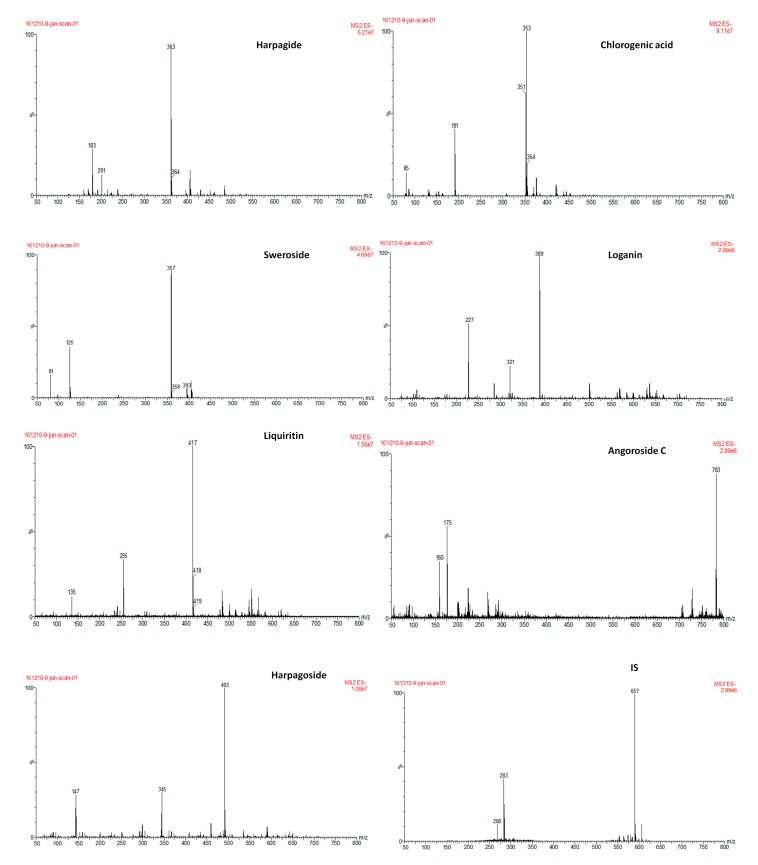
Product ion mass spectra of the analytes and IS.

Selectivity: The selectivity of the method was assessed by comparing the chromatograms of blank plasma samples collected from six different individual rats, plasma samples spiked with the analytes and IS and plasma samples obtained from a rat at 60 min after oral administration of the SYD extract (Figure 4).

LLOQ: Calibration curves were obtained by plotting the measured peak area ratios of analytes to IS. Standard curves representing the peak area ratios versus analyte concentrations were described in the form of *y* = a + b*x* [*f* = (Mi/Ai)/(Ms/As)]. The LLOQ for each analyte was the lowest concentration with a signal to noise of ≥10 that could be quantitatively determined with a precision and accuracy of ≤15% based on the analysis of six replicates of each sample.

Accuracy and Precision: Precision and accuracy were evaluated at three concentrations. The intra-day accuracy and precision were estimated by analyzing six QC samples at each concentration level during the same day, while the inter-day accuracy and precision were assessed on six consecutive days. The different precisions were defined as the relative standard deviation (RSD, %), which should be <15%. The accuracy was expressed by the relative error (RE, %).

Extraction Recovery and Matrix Effect: The extraction recovery of five analytes at three QC levels was evaluated by comparing the peak areas obtained from the extracted QC samples with those obtained from reference standards spiked into post-extracted blank plasma. The matrix effect was determined by comparing the peak response of theanalytes in plasma samples with those of the pure standardsprepared in the mobile phase.Six replicates of each concentration of quality control samples were prepared.

Stability: The stability of the seven reference analytes (harpagide, chlorogenic acid, sweroside, loganin, liquiritin, angoroside C and harpagoside) in rat plasma was evaluated with QC samples at three concentrations, each in six replicates, under different storage and processing conditions, including the stability of plasma samples kept at room temperature for 6 h, at −20 °C for 14 days and after three freeze-thaw cycles; and of the post-preparation sample in the auto-sampler at 4 °C for 24 h. Stability was evaluated by comparing the mean concentration of the stored QC samples with the mean concentration of freshly prepared QC samples.

### 3.7. Pharmacokinetic Study

The SYD was decomposed into 15 groups of compatibility combinations (LJ, SN, AS, GU, LJ + SN, LJ + AS, LJ + GU, SN + AS, SN + GU, AS + GU, LJ + SN + AS, LJ + SN + GU, LJ + AS + GU, SN + AS + GU and SYD). The 15 combinations were weighed according to the proportion of 3:3:2:1 with appropriate amounts and prepared by the method described in “Section 3.2”. Six male SD rats (body weight 250 ± 10 g) of each compatibility group were used in the pharmacokinetic study. After 12-h fast, rats of the different groups were orally administered the respective extract dissolved in saline (Table 4). Venous blood (0.45 mL) was obtained from the orbit vein and collected in heparin-pretreated polypropylene centrifuge tubes at 0.083 h, 0.17 h, 0.25 h, 0.50 h, 0.75 h, 1.0 h, 1.5 h, 2.0 h, 3.0 h, 4.0 h, 6.0 h, 8.0, 12 h and 24 h post-dose. The plasma was separated by centrifugation at 8000 r/min for 10 min and then stored at −20 °C until analysis. The amount of each analyte in the plasma was estimated by UPLC-MS/MS analysis as described in Section 3.5.

### 3.8. Pharmacokinetic Data Analysis

Non-compartmental methods using Phoenix WinNonlin 7.0 software provided by the Beijing University of Chinese Medicine were used to analyze plasma concentration versus time profiles and to estimate the following pharmacokinetic parameters: terminal elimination half-lives (*t*_1/2_), area under the plasma concentration versus time curve from zero to last sampling time (AUC), and the mean residence time (MRT). The peak plasma concentration (*C*_max_) and time to reach peak plasma concentration (*T*_max_) for each dose were read directly from the observed individual plasma concentration-time data.

## 4. Conclusions

We describe here the development and validation of a rapid and sensitive UPLC-MS/MS method for the simultaneous analysis of harpagide, chlorogenic acid, sweroside, loganin, liquiritin, angoroside C and harpagoside in rat plasma. The method was successfully applied to the pharmacokinetic study of these seven analytes in rat plasma samples after oral administration of SYD extract and the corresponding single and combined TCMs. The findings indicated that the pharmacokinetic properties of these seven detected analytes were dynamically changed due to counteraction among the coexisting components. The pharmacokinetic study of complex TCM prescription should be performed in an integral form. Our results also provide useful information for further clinical applications of Chinese traditional formulations.

## Figures and Tables

**Table 1:** Ultra-performance liquid chromatography-tandem mass spectrum (UPLC-MS/MS) transitions and parameters for the detection of the analytes and internal standard (IS).

t/min	Analyte	[M − H]^−^ (*m*/*z*)	Quantitative Ions (*m*/*z*)	Qualitative Ion (*m*/*z*)	Cone Voltage	Collision Energy
5.73	Harpagide	363.22	183.11	201.07	2	14
8.79	Chlorogenic acid	353.22	191.05	85.02	42	16
11.12	Sweroside	357.22	125.07	81.05	54	14
11.37	Loganin	389.22	227.12	321.1	56	4
13.54	Liquiritin	417.29	255.1	135.02	10	18
19.92	Angoroside C	783.54	175.05	160.03	2	32
23.76	Harpagoside	493.35	147.03	345.13	2	22
22.58	IS	591.45	283.15	268.13	50	16

**Table 2:** The accuracy (intra- and inter- day), precision (intra- and inter- day), recovery, matrix effects and stability for 7 compounds in rat plasma (*n* = 18, 6 replicates per day for 3 days).

Compound	Concentration (ng/mL)	Accuracy (RE, %)		Precision (RSD, %)		Recovery		Matrix Effects		Auto-Sampler 4 °C for 24 h		At room Temperature for 4 h		At −20 °C for 2 Weeks		Freeze-Thaw Cycles	
		Intra-Day	Inter-Day	Intra-Day	Inter-Day	Accuracy (%)	Precision (%)	Accuracy (%)	Precision (%)	Mean (%)	RSD (%)	Mean (%)	RSD (%)	Mean (%)	RSD (%)	Mean (%)	RSD (%)
Harpagide	36.021	106.8	109.5	8.1	3.1	89.8	2.5	81.9	2.4	95.2	4.2	104.1	2.5	96.3	2.9	93.0	2.4
	346.908	101.6	100.3	2.4	2.2	93.1	4.6	82.1	1.2	98.4	2.5	101.7	4.3	96.7	8.3	103.2	9.1
	1012.666	107.4	108.6	5.1	3.3	88.7	4.1	82.9	3.1	99.7	4.0	102.1	4.8	98.1	2.4	104.5	3.6
Chlorogenic acid	61.600	102.5	91.4	2.6	3.5	88.1	6.4	92.3	6.6	104.2	3.7	104.5	0.8	96.4	2.6	97.2	2.7
	593.260	98.6	96.9	4.6	0.9	86.3	4.7	98.0	2.2	101.9	2.5	100.2	4.4	99.5	5.3	95.1	0.9
	1731.796	104.1	100.3	3.0	3.2	94.6	8.6	87.6	10.6	96.5	5.7	97.4	1.8	98.2	3.8	96.9	4.1
Sweroside	119.394	104.8	94.8	3.3	1.5	87.3	4.5	85.8	9.2	97.8	4.1	99.5	1.8	97.8	4.1	103.6	3.5
	1149.863	99.8	102.6	3.5	4.5	99.5	6.0	90.4	5.7	103.4	1.9	96.6	2.8	100.5	4.2	104.7	2.4
	3356.586	92.5	105.0	2.3	4.4	98.2	8.1	90.3	7.7	104.5	3.2	93.4	6.1	101.2	5.4	95.2	8.4
Loganin	35.670	95.4	108.1	5.5	4.6	90.0	7.6	80.6	4.5	100.8	4.5	98.1	5.1	104.9	4.2	99.5	2.9
	343.533	99.5	102.0	5.2	7.8	99.5	6.8	81.1	4.1	98.6	6.7	104.3	5.1	103.1	5.0	97.9	2.6
	1002.812	107.8	108.7	1.3	1.3	96.1	8.2	85.0	5.8	99.1	7.5	102.3	7.0	104.5	4.5	104.8	6.1
Liquiritin	31.832	102.9	105.7	3.3	1.9	88.8	2.3	87.9	2.6	103.2	1.6	95.4	2.0	102.4	1.1	104.2	2.5
	306.572	99.7	97.3	4.4	2.7	93.8	3.0	87.5	1.3	104.5	1.5	99.0	6.4	98.3	3.4	96.2	4.7
	894.920	100.1	99.7	4.7	1.3	93.0	4.6	88.5	3.9	96.7	1.6	96.3	1.6	97.7	1.5	95.3	3.9
Angoroside C	27.215	104.4	103.5	3.8	3.7	97.4	7.1	91.3	4.0	95.9	3.6	98.1	2.3	98.2	4.7	99.4	3.2
	262.099	98.9	98.2	5.1	4.4	93.3	5.0	94.1	4.1	99.1	3.3	99.5	4.0	96.1	8.5	96.9	3.1
	765.098	104.4	97.8	5.3	2.7	91.7	2.4	93.5	5.5	98.5	1.6	100.4	1.3	99.4	3.0	100.7	2.6
Harpagoside	12.628	110.0	90.1	5.2	2.7	93.1	13.6	84.9	9.7	97.7	4.0	104.3	6.6	97.5	2.2	104.7	4.1
	121.615	104.1	99.0	5.7	2.7	93.7	4.3	83.4	3.4	99.0	9.4	101.2	2.5	98.6	9.8	100.6	7.8
	355.008	95.7	95.0	3.5	2.5	89.3	7.5	84.1	4.2	98.6	6.6	104.5	9.3	104.3	5.8	103.4	5.5
IS	36.021	92.1	96.3	1.9	2.8	84.9	1.0	111.9	3.4	96.2	2.1	98.2	5.3	96.3	3.2	96.3	4.3
	346.908	103.5	98.2	2.1	3.6	82.9	3.2	113.6	3.6	103.2	4.3	96.3	3.2	103.2	4.6	95.1	3.6
	1012.666	104.0	96.3	3.8	4.5	86.8	0.8	105.9	1.4	105.1	3.6	103.1	1.6	104.3	1.2	102.3	2.8

**Table 3:** Pharmacokinetic parameters of the seven compounds tested in plasma samples collected from rats orally administered fifteen compatibility combination extracts of SYD (mean ± SD, *n* = 6 replicates).

Pharmacokinetic Parameters *	SYD	LJ	AS	SN	GU	LJ + SN + AS	LJ + SN + GU	SN + AS + GU	LJ + AS + GU	LJ + SN	LJ + AS	LJ + GU	SN + AS	SN + GU	AS + GU
Harpagide															
AUC_(0–*t*)_ (h μg/mL)	1.57 ± 0.25	/	/	2.77 ± 0.34	/	1.58 ± 0.19	1.45 ± 0.26	1.72 ± 0.22	/	1.41 ± 0.17	/	/	1.61 ± 0.33	1.16 ± 0.15	/
AUC_(0–∞)_ (h μg/mL)	2.61 ± 1.73	/	/	2.94 ± 0.37	/	2.62 ± 1.83	2.67 ± 1.81	2.76 ± 1.82	/	2.50 ± 1.86	/	/	2.76 ± 1.69	2.39 ± 1.85	/
MRT_(0–*t*)_ (h)	4.92 ± 0.56	/	/	4.26 ± 0.46	/	4.82 ± 0.56	5.12 ± 0.59	4.58 ± 0.49	/	5.18 ± 0.19	/	/	5.17 ± 0.62	5.98 ± 0.42	/
MRT_(0–∞)_ (h)	52.75 ± 10.42	/	/	6.39 ± 2.61	/	52.09 ± 101.60	62.09 ± 10.74	50.90 ± 99.45	/	55.30 ± 10.97	/	/	58.81 ± 10.17	65.43 ± 10.77	/
*t*_1/2_ (h)	7.72 ± 1.78	/	/	6.70 ± 2.94	/	6.30 ± 0.99	8.34 ± 1.93	7.12 ± 2.34	/	7.14 ± 0.84	/	/	7.93 ± 2.55	8.79 ± 2.77	/
*T*_max_ (h)	2.00 ± 0.00	/	/	1.00 ± 0.00	/	1.58 ± 0.20	1.58 ± 0.20	1.67 ± 0.26	/	1.17 ± 0.26	/	/	1.67 ± 0.41	1.42 ± 0.38	/
*C*_max_ (μg/mL)	0.67 ± 0.16	/	/	1.12 ± 0.12	/	0.60 ± 0.12	0.62 ± 0.11	0.75 ± 0.15	/	0.59 ± 0.11	/	/	0.55 ± 0.15	0.35 ± 0.12	/
Chlorogenic acid															
AUC_(0–*t*)_ (h μg/mL)	2.54 ± 0.28	3.94 ± 2.12	1.36 ± 0.12	/	/	2.55 ± 0.64	2.19 ± 0.48	2.23 ± 0.45		2.80 ± 1.16	2.82 ± 0.75	1.78 ± 0.29	/	/	1.15 ± 0.34
AUC_(0–∞)_ (h μg/mL)	3.65 ± 0.82	5.04 ± 2.98	2.13 ± 0.67	/	/	3.69 ± 0.49	3.41 ± 0.64	3.36 ± 0.781		4.25 ± 2.31	3.77 ± 1.22	2.93 ± 1.00	/	/	1.98 ± 0.45
MRT_(0–*t*)_ (h)	3.28 ± 0.50	3.12 ± 0.75	1.56 ± 0.67	/	/	3.19 ± 0.32	3.44 ± 0.29	3.43 ± 0.51		3.48 ± 0.86	3.17 ± 0.75	3.85 ± 0.88	/	/	1.78 ± 0.39
MRT_(0–∞)_ (h)	52.63 ± 53.81	24.71±8.81	4.28 ± 2.23	/	/	59.47 ± 63.22	69.29 ± 73.59	60.05 ± 62.56		46.84 ± 43.37	37.64 ± 36.10	65.00 ± 64.58	/	/	3.36 ± 2.00
*t*_1/2_ (h)	8.08 ± 1.67	9.39 ± 2.87	3.54 ± 0.98	/	/	6.73 ± 1.70	8.34 ± 1.07	7.95 ± 2.46		7.82 ± 2.60	7.14 ± 1.59	8.15 ± 2.49	/	/	2.78 ± 0.53
*T*_max_ (h)	1.67 ± 0.26	1.11 ± 0.74	0.89 ± 0.33	/	/	1.58 ± 0.38	1.67 ± 0.26	1.58 ± 0.38		1.36 ± 1.03	1.36 ± 0.69	1.18 ± 0.78	/	/	0.55 ± 0.48
*C*_max_ (μg/mL)	1.22 ± 0.08	2.41 ± 0.83	0.39 ± 0.11	/	/	1.38 ± 0.37	1.28 ± 0.51	1.21 ± 0.47		1.74 ± 0.75	1.67 ± 0.48	1.07 ± 0.19	/	/	0.29 ± 0.21
Sweroside															
AUC_(0–*t*)_ (h μg/mL)	4.49 ± 0.28	6.90 ± 2.66	/	/	/	8.16 ± 1.95	7.11 ± 2.09	/	7.23 ± 2,.18	7.14 ± 1.16	6.49 ± 1.18	7.02 ± 1.96	/	/	/
AUC_(0–∞)_ (h μg/mL)	4.53 ± 0.29	7.86 ± 4.08	/	/	/	9.58 ± 2.76	7.48± 1.96	/	7.59 ± 1.95	7.53 ± 1.82	6.72 ± 1.16	7.26 ± 1.96	/	/	/
MRT_(0–*t*)_ (h)	3.30 ± 0.39	4.46 ± 1.20	/	/	/	4.26 ± 1.21	3.72 ± 0.42	/	3.70 ± 0.45	3.61 ± 0.57	3.67 ± 0.64	3.55 ± 0.64	/	/	/
MRT_(0–∞)_ (h)	3.53 ± 0.29	7.50 ± 5.21	/	/	/	10.90 ± 7.98	6.54 ± 5.01	/	6.38 ± 4.85	6.18 ± 3.35	4.87 ± 1.37	4.90 ± 1.42	/	/	/
*t*_1/2_ (h)	4.14 ± 1.46	7.39 ± 6.33	/	/	/	4.50 ± 2.42	5.24 ± 2.95	/	8.92 ± 10.01	6.10 ± 4.48	6.69 ± 4.91	4.49 ± 1.49	/	/	/
*T*_max_ (h)	1.33 ± 0.49	0.96 ± 0.58	/	/	/	1.42 ± 0.56	1.38 ± 0.54	/	1.38 ± 0.54	1.04 ± 0.62	1.04 ± 0.62	1.13 ± 0.52	/	/	/
*C*_max_ (μg/mL)	1.97 ± 0.70	2.92 ± 1.77	/	/	/	2.84 ± 0.73	2.38 ± 0.79	/	2.47 ± 0.85	2.63 ± 0.86	2.24 ± 0.76	2.90 ± 1.05	/	/	/
Loganin															
AUC_(0–*t*)_ (h μg/mL)	1.98 ± 1.38	1.52 ± 0.11	/	/	/	1.16 ± 0.78	1.84 ± 0.45	/	1.66 ± 0.12	1.79 ± 0.44	1.72 ± 0.31	1.60 ± 0.07	/	/	/
AUC_(0–∞)_ (h μg/mL)	2.17 ± 1.40	1.67 ± 0.34	/	/	/	1.18 ± 0.77	5.51 ± 2.02	/	5.35± 1.94	5.47 ± 2.06	5.40 ± 1.94	5.51 ± 2.00	/	/	/
MRT_(0–*t*)_ (h)	4.71 ± 1.34	3.65 ± 1.03	/	/	/	2.50 ± 0.70	7.72 ± 1.48	/	8.06 ± 0.78	7.79 ± 1.38	7.93 ± 1.33	8.17 ± 0.62	/	/	/
MRT_(0–∞)_ (h)	8.87 ± 8.10	6.74 ± 6.00	/	/	/	4.07 ± 3.93	79.29 ± 40.84	/	81.92 ± 41.27	79.98 ± 40.25	80.89 ± 41.48	88.48 ± 42.11	/	/	/
*t*_1/2_ (h)	7.45 ± 3.39	6.71 ± 4.19	/	/	/	6.25 ± 4.29	6.83 ± 2.11	/	7.91 ± 2.17	9.39 ± 2.60	7.89 ± 2.42	8.07 ± 3.65	/	/	/
*T*_max_ (h)	1.42 ± 0.38	1.25 ± 0.59	/	/	/	1.50 ± 0.45	1.50 ± 0.45	/	1.50 ± 0.45	1.54 ± 0.56	1.33 ± 0.52	1.25 ± 0.59	/	/	/
*C*_max_ (μg/mL)	0.77 ± 0.46	1.14 ± 0.52	/	/	/	0.66 ± 0.483	0.72 ± 0.45	/	0.54 ± 0.17	0.68 ± 0.1	0.66 ± 0.35	0.69 ± 0.44	/	/	/
Liquiritin															
AUC_(0–*t*)_ (h μg/mL)	0.29 ± 0.21	/	/	/	0.19 ± 0.00	/	0.23 ± 0.16	0.21 ± 0.11	0.22 ± 0.13	/	/	0.18 ± 0.06	/	0.18 ± 0.05	0.22 ± 0.08
AUC_(0–∞)_ (h μg/mL)	0.34 ± 0.21	/	/	/	0.19 ± 0.00	/	0.26 ± 0.17	0.24 ± 0.12	0.26 ± 0.14	/	/	0.20 ± 0.06	/	0.21 ± 0.05	0.24 ± 0.08
MRT_(0–*t*)_ (h)	2.89 ± 0.92	/	/	/	1.16 ± 0.01	/	2.50 ± 0.67	2.55 ± 0.57	2.41 ± 0.63	/	/	2.53 ± 0.56	/	2.48 ± 0.46	2.30 ± 0.63
MRT_(0–∞)_ (h)	11.29 ± 7.16	/	/	/	1.19 ± 0.01	/	16.34 ± 12.86	16.05 ± 10.97	16.00 ± 11.76	/	/	13.52 ± 11.03	/	12.44 ± 9.87	11.09 ± 8.58
*t*_1/2_ (h)	13.40 ± 5.59	/	/	/	4.41 ± 0.59	/	13.40 ± 5.15	11.58 ± 5.01	13.69 ± 4.69	/	/	12.20 ± 7.30	/	7.81 ± 5.31	9.28 ± 5.92
*T*_max_ (h)	0.88 ± 0.31	/	/	/	0.08 ± 0.00	/	0.88 ± 0.31	1.00 ± 0.27	0.79 ± 0.29	/	/	0.56 ± 0.56	/	0.42 ± 0.53	0.60 ± 0.50
*C*_max_ (μg/mL)	0.21 ± 0.17	/	/	/	0.27 ± 0.00	/	0.19 ± 0.17	0.163 ± 0.14	0.21 ± 0.17	/	/	0.14 ± 0.07	/	0.15 ± 0.07	0.20 ± 0.13
Angoroside C															
AUC_(0–*t*)_ (h μg/mL)	0.30 ± 0.29	/	/	0.09 ± 0.03	/	0.18 ± 0.13	0.22 ± 0.09	0.22 ± 0.17	/	0.39 ± 0.46	/	/	0.45 ± 0.03	0.41 ± 0.06	/
AUC_(0–∞)_ (h μg/mL)	0.31 ± 0.29	/	/	0.11 ± 0.02	/	0.20 ± 0.13	0.23 ± 0.19	0.23 ± 0.18	/	0.40 ± 0.46	/	/	0.46 ± 0.03	0.42 ± 0.08	/
MRT_(0–*t*)_ (h)	3.01 ± 0.69	/	/	3.03 ± 0.37	/	3.05 ± 0.70	2.95 ± 0.82	3.01 ± 0.72	/	2.88 ± 1.23	/	/	2.85 ± 1.25	2.86 ± 1.21	/
MRT_(0–∞)_ (h)	7.01 ± 5.95	/	/	18.11 ± 3.41	/	8.16 ± 6.76	7.88 ± 6.88	7.79 ± 6.70	/	5.22 ± 3.50	/	/	5.01 ± 3.31	4.99 ± 3.19	/
*t*_1/2_ (h)	11.38 ± 6.34	/	/	10.68 ± 6.13	/	11.82 ± 6.58	11.74 ± 6.52	11.59 ± 6.42	/	10.35 ± 5.06	/	/	10.03 ± 4.72	9.91 ± 4.62	/
*T*_max_ (h)	1.38 ± 0.44	/	/	1.25 ± 0.59	/	1.38 ± 0.44	1.29 ± 0.46	1.38 ± 0.44	/	1.07 ± 0.54	/	/	1.03 ± 0.49	1.11 ± 0.52	/
*C*_max_ (μg/mL)	0.18 ± 0.20	/	/	005 ± 0.03	/	0.14 ± 0.13	0.15 ± 0.15	0.15 ± 0.14	/	0.27 ± 0.33	/	/	0.40 ± 0.02	0.33 ± 0.05	/
Harpagoside															
AUC_(0–*t*)_ (h μg/mL)	0.12 ± 0.09	/	/	0.31 ± 0.11	/	0.11 ± 0.08	0.15 ± 0.07	0.07 ± 0.01	/	0.06 ± 0.01	/	/	0.10 ± 0.07	0.08 ± 0.01	/
AUC_(0–∞)_ (h μg/mL)	0.14 ± 0.09	/	/	0.32 ± 0.11	/	0.11 ± 0.08	0.19 ± 0.11	0.10 ± 0.01	/	0.09 ± 0.01	/	/	0.13 ± 0.09	0.11 ± 0.08	/
MRT_(0–*t*)_ (h)	3.92 ± 1.31	/	/	4.75 ± 1.17	/	3.16 ± 0.68	3.17 ± 0.59	5.13 ± 1.98	/	5.32 ± 1.39	/	/	4.51 ± 2.04	5.72 ± 2.55	/
MRT_(0–∞)_ (h)	19.81 ± 8.74	/	/	5.69 ± 3.19	/	13.02 ± 19.90	22.65 ± 31.10	46.65 ± 77.56	/	46.27 ± 70.72	/	/	41.86 ± 67.29	47.48 ± 78.22	/
*t*_1/2_ (h)	11.20 ± 2.64	/	/	3.34 ± 3.36	/	9.79 ± 5.03	13.27 ± 4.59	15.53 ± 1.54	/	15.75 ± 1.19	/	/	16.61 ± 1.40	18.32 ± 6.90	/
*T*_max_ (h)	1.50 ± 0.00	/	/	1.08 ± 0.47	/	1.63 ± 0.49	1.79 ± 0.51	0.83 ± 0.13	/	1.67 ± 0.41	/	/	1.42 ± 0.38	1.25 ± 0.59	/
*C*_max_ (μg/mL)	0.07 ± 0.07	/	/	0.11 ± 0.05	/	0.05 ± 0.00	0.08 ± 0.00	0.04 ± 0.00	/	0.03 ± 0.00	/	/	0.07 ± 0.01	0.05 ± 0.01	/

**Table 4:** The dose of different compatibility combinations for oral administration.

Group	Extract (g·kg^−1^)	Herbs (g·kg^−1^)	Group	Extract (g·kg^−1^)	Herbs (g·kg^−1^)	Group	Extract (g·kg^−1^)	Herbs (g·kg^−1^)
SYD	8.25	15	JX	4.5	10	GU + AS	2.43	5
LJ	2.15	5	JD	3.29	8.33	LJ + SN + AS	5.43	13.33
SN	2.49	5	JG	2.14	6.67	LJ + SN + GU	4.73	11.67
AS	1.29	3.33	XD	3.98	8.33	LU + GU + AS	4.01	10
GU	0.68	1.67	XG	3.38	6.67	SN + AS + GU	4.28	10
